# Sleep deprivation prevents counterregulatory adaptation to recurrent hypoglycaemia

**DOI:** 10.1007/s00125-022-05702-9

**Published:** 2022-04-21

**Authors:** Svenja Meyhöfer, Katharina Dembinski, Bernd Schultes, Jan Born, Britta Wilms, Hendrik Lehnert, Manfred Hallschmid, Sebastian M. Meyhöfer

**Affiliations:** 1 Institute for Endocrinology & Diabetes, University of Lübeck, Lübeck, Germany; 2 German Center for Diabetes Research (DZD), München-Neuherberg, Germany; 3 Department of Internal Medicine 1, Endocrinology & Diabetes, University of Lübeck, Lübeck, Germany; 4 Metabolic Center St Gallen, FriendlyDocs Ltd, St Gallen, Switzerland; 5 Deparment of Medical Psychology and Behavioral Neurobiology, University of Tübingen, Tübingen, Germany; 6 Institute for Diabetes Research and Metabolic Diseases of the Helmholtz Center Munich at the University of Tübingen, Tübingen, Germany; 7 University of Salzburg, Salzburg, Austria

**Keywords:** Diabetes complications, Hormonal counterregulation, Hypoglycaemia unawareness, Metabolic memory, Recurrent hypoglycaemia, Sleep deprivation

## Abstract

**Aims/hypothesis:**

Attenuated counterregulation after recurrent hypoglycaemia is a major complication of diabetes treatment. As there is previous evidence for the relevance of sleep in metabolic control, we assessed the acute contribution of sleep to the counterregulatory adaptation to recurrent hypoglycaemia.

**Methods:**

Within a balanced crossover design, 15 healthy, normal-weight male participants aged 18–35 years underwent three hyperinsulinaemic–hypoglycaemic clamps with a glucose nadir of 2.5 mmol/l, under two experimental conditions, sleep and sleep deprivation. Participants were exposed to two hypoglycaemic episodes, followed by a third hypoglycaemic clamp after one night of regular 8 h sleep vs sleep deprivation. The counterregulatory response of relevant hormones (glucagon, growth hormone [GH], ACTH, cortisol, adrenaline [epinephrine] and noradrenaline [norepinephrine]) was measured, and autonomic and neuroglycopenic symptoms were assessed.

**Results:**

Sleep deprivation compared with sleep dampened the adaptation to recurrent hypoglycaemia for adrenaline (*p*=0.004), and this pattern also emerged in an overall analysis including adrenaline, GH and glucagon (*p*=0.064). After regular sleep, the counterregulatory responses of adrenaline (*p*=0.005), GH (*p*=0.029) and glucagon (*p*=0.009) were attenuated during the 3rd clamp compared with the 1st clamp, but were preserved after sleep deprivation (all *p*>0.225). Neuroglycopenic and autonomic symptoms during the 3rd clamp compared with the 1st clamp were likewise reduced after sleep (*p*=0.005 and *p*=0.019, respectively). In sleep deprivation, neuroglycopenic symptoms increased (*p*=0.014) and autonomic symptoms were unchanged (*p*=0.859).

**Conclusions/interpretation:**

The counterregulatory adaptation to recurrent hypoglycaemia is compromised by sleep deprivation between hypoglycaemic episodes, indicating that sleep is essential for the formation of a neurometabolic memory, and may be a potential target of interventions to treat hypoglycaemia unawareness syndrome.

**Graphical abstract:**

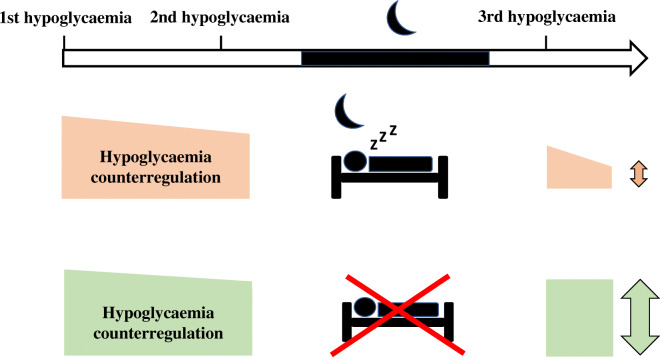

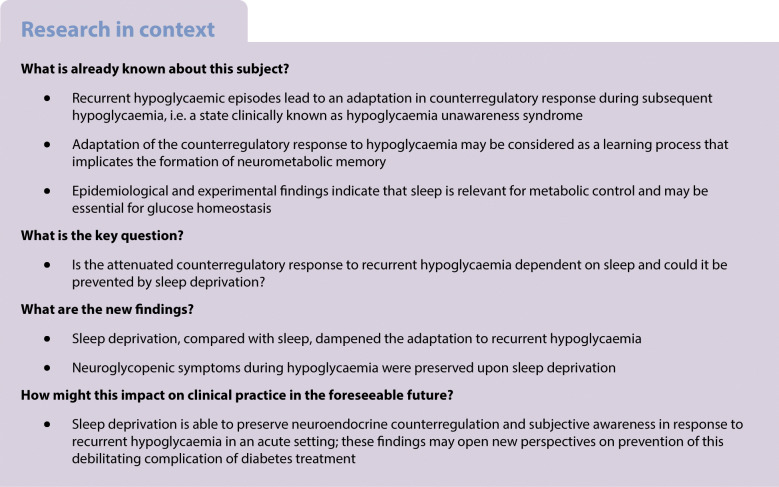



## Introduction

Hypoglycaemia is a serious complication in the treatment of diabetes, and can compromise compliance because of concerns about loss of consciousness, seizure or even death [1]. Physiologically, hypoglycaemia triggers a counterregulatory stress response including release of adrenaline (epinephrine), glucagon, cortisol, growth hormone (GH) and other hormones, together with induction of autonomous and neuroglycopenic symptoms. As a consequence, blood glucose increases via glycogenolysis, gluconeogenesis and inhibition of glucose utilisation [2]. Hypoglycaemia counterregulation is mediated by the sympathetic nervous system and hormonal changes. It is a complex process regulated by distinct nuclei within the hypothalamus to preserve the body’s energy supply. Patients with diabetes frequently experience recurrent hypoglycaemic episodes, leading to adaptation of the counterregulatory response during subsequent hypoglycaemia, i.e. attenuated hormonal reactions and dampened autonomous and neuroglycopenic symptoms. It has been assumed that the dorsal midline thalamus not only contributes to hypoglycaemia counterregulation but also to habituation during recurrent hypoglycaemia [3]. Notably, even mild hypoglycaemic episodes can trigger habituation, and a single hypoglycaemic episode can reduce hypoglycaemia awareness, partly due to a shift of the glycaemic threshold for activation of the sympathetic nervous system when plasma glucose levels are low [4]. The resulting hypoglycaemia-associated autonomic failure contributes to the clinical syndrome of hypoglycaemia unawareness [5], which represents a major challenge in the treatment of diabetes [1]. Hypoglycaemia unawareness can be reversed by preventing hypoglycaemia over a longer period of time [6].

Adaptation of the counterregulatory response to hypoglycaemia can be considered as a learning process that implicates the formation of a neurometabolic memory; because of its clinical relevance, a better understanding of the underlying mechanisms of plasticity is needed to develop strategies against hypoglycaemia unawareness syndrome. One potential modulator of metabolic memory might be sleep, which is well known to essentially underpin processes of memory consolidation. The beneficial memory effect of sleep in the cognitive domain, e.g. on declarative and procedural memory, is well known [7–9]. Hippocampus-dependent declarative memory is consolidated during sleep [10], a state during which groups of neurons in the hippocampus, basal forebrain and anterior hypothalamus raise their activity intermittently [11]. Interestingly, these neurons are also involved in the regulation of energy and glucose homeostasis [4]. Epidemiological and experimental findings indicate that sleep is relevant for metabolic control [12], and that deep sleep, which is characterised by slow oscillatory brain activity, might be essential for glucose homeostasis [13]. However, the underpinnings of metabolic memory, as reflected by counterregulatory adaptation to recurrent hypoglycaemia, and the respective contribution of sleep-related processes, are still unknown. Against this background, we investigated counterregulatory adaptation, i.e. the attenuated counterregulatory response to recurrent hypoglycaemia, as a clinically relevant model of neurometabolic memory, and hypothesised that it is dependent on sleep, and thus is prevented by sleep deprivation.

## Methods

### Participants

Fifteen healthy normal-weight men aged 18–35 years (mean ± SEM 22.4 ± 0.97 years), with a BMI between 20.0 and 24.9 kg/m^2^ (mean ± SEM 22.6 ± 0.45 kg/m^2^), were enrolled in the study. Assuming a medium effect size of *f* = 0.7 for the effect of sleep vs sleep deprivation based on previous experiments on the effect of sleep loss on glucose homeostasis [14], power analyses using G*Power, version 3.1 (provided by Heinrich Heine University Düsseldorf) revealed that a sample size of *N* = 13 was necessary to detect an effect at a significance level of *α* = 0.05. Exclusion criteria were: smoking, current medication of any kind, elevated consumption of alcohol (>50 g per day) and/or caffeine (>300 mg per day), use of prescription drugs, shift work, travel across time zones during the previous 4 weeks, short habitual sleep duration (<6 h per day), blood donation within the past 8 weeks, psychological or physical illnesses of any kind, diabetes mellitus in first-degree relatives, as well as abnormal findings in a physical examination including routine laboratory testing. The study protocol was approved by the Ethics Committee on Research involving Humans at the University of Lübeck, and all participants gave written informed consent prior to participation.

### Study design

**Fig. 1:**
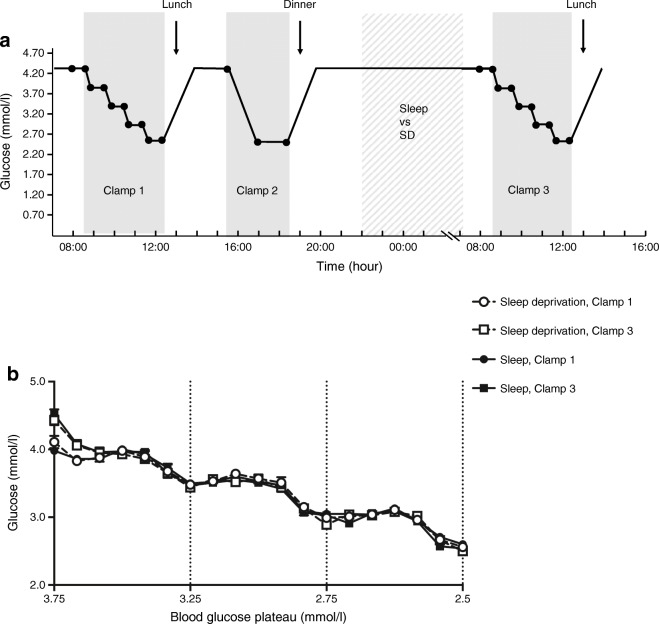
(**a**) Study design, showing glucose levels over the time period, with timing of meals and clamps. (**b**) Blood glucose concentrations during clamps 1 and 3 under both conditions. SD, Sleep deprivation

All participants took part in two experimental sessions scheduled in randomised order. Each session included a complete day (1st day) followed by a night of either total sleep deprivation or 8 h of regular sleep, and the subsequent morning (2nd day). On the 1st day, hyperinsulinaemic–hypoglycaemic clamps were performed both in the morning (clamp 1) and in the afternoon (clamp 2). On the 2nd day, a third hyperinsulinaemic–hypoglycaemic clamp (clamp 3) was performed in the morning (Fig. 1a). The time interval between experimental sessions was at least four weeks but no more than ten weeks.

### Study procedure

Participants arrived in the fasted state for all experiments, which were performed in a soundproof sleep laboratory equipped with an infrared camera to monitor participants during the entire experiment. The first experimental day started at 07:00 hours. An 18G intravenous catheter was inserted into a vein of the participant’s left forearm, and a 20G catheter was inserted into a vein of the right forearm. Arterialised blood was obtained by heating the hand and forearm in a warm-air box set to 55–60°C, following a standard approach [15]. Before starting the 1st clamp, two blood samples were taken at 08:10 hours and 08:40 hours to determine individual baseline values. A stepwise hypoglycaemic clamp was initiated at 08:45 hours (clamp 1). At 15:30 hours on the same day, a simplified clamp (clamp 2) was performed for induction of an adaptation in hypoglycaemia counterregulation. At 08:45 hours on the subsequent day, another stepwise clamp (clamp 3) was performed.

The procedure used for the stepwise hypoglycaemic clamp adhered to a standardised protocol previously described in detail [16]. In short, 1.5 mU kg^−1^ min^−1^ human insulin (Insuman Rapid, 40 U/ml, Sanofi-Aventis, Germany) was continuously infused into the participant’s right arm by infusion pump. Venous glucose was measured at intervals of 2–5 min, and the infusion rate of a 20% glucose solution was adjusted to reach the target glucose values. Over 30 min intervals, blood glucose was lowered to or maintained at the target blood glucose plateau (3.75, 3.25, 2.75, and 2.5 mmol/l.; Fig. 1b). Blood samples for hormone analyses were taken during steady state of the respective plateau. After clamp 1 on the 1st day, a standardised lunch was served at 12:45 hours. To enhance the adaptation of counterregulatory responses, the second hypoglycaemic episode was induced at 15:30 hours by clamp 2 following a simplified clamp protocol. Blood glucose was decreased to 2.5 mmol/l during the first 60 min of clamp 2, and maintained at nadir plateau for another 60 min. A standardised dinner was served after clamp 2 at 19:00 hours. In the sleep condition, participants were prepared for polysomnography at 21:00 hours, and lights were switched off at 23:00 hours. They were woken up at 07:00 hours. In the sleep deprivation condition, participants stayed awake and spent their time under constant supervision with a fixed repertoire of movies and games. Under both conditions, clamp 3 was performed at 08:45 hours using the same protocol as clamp 1. Participants avoided energy intake in excess of the provided meals, and refrained from strenuous physical activity and coffee intake during the entire experiment.

### Blood samples and assays

Blood glucose was determined online using a BIOSEN C-Line glucose analyser (EKF Diagnostics, Germany), with a mean value of 12 mmol/l, and a within-assay variation below 1.5% according to the manufacturer. All other values were determined using serum or plasma samples stored at −80°C. Concentrations of GH, ACTH and cortisol were assessed by immunoassay (Immulite 2000, Siemens Healthcare Diagnostics, UK) with detection limits and mean within-assay CV, respectively, of 0.01 ng/ml and 5.3–6.5% for GH, 9 pg/ml and 3.1–9.6% for ACTH, and 5.5 nmol/l and ≤7.4% for cortisol. Glucagon concentrations were measured by radioimmunoassay (Euro Diagnostica, Sweden), with a mean within-assay CV of <2.5%. Concentrations of adrenaline and noradrenaline (norepinephrine) were determined by HPLC (Chromsystems, Germany).

### Symptoms

Participants’ subjective symptoms were assessed using standardised symptom lists as described by Mitrakou et al [17]. This questionnaire included typical subjective symptoms of hypoglycaemia (dizziness, tingling, blurred vision, difficulty in thinking, faintness) as neuroglycopenic symptoms, as well as anxiety, palpitations, hunger, sweating, irritability and tremor as autonomic symptoms; the symptoms were rated on a scale from 0 (not at all) to 9 (very high). The sum of each of these subcategories constituted the respective symptom score.

### Sleep recordings

For standard polysomnography, electrodes were attached to the scalp for electroencephalographic recordings; above, below, and beside the eyes for horizontal and vertical electrooculogram; and on the chin for electromyogram (two electrodes). Recordings were performed using a Nihon Kohden amplifier (EEG 9200G series, Nihon Kohden, Germany). Data were scored offline according to standard criteria [18], and the following sleep variables were determined: total sleep time, wake time after sleep onset, i.e. time in ‘awake stage’ after starting to sleep, time spent in non-rapid eye movement (REM) sleep stages 1 and 2, time in slow-wave sleep (SWS), and time in REM sleep (all in terms of absolute duration and as a percentage of total sleep time).

### Statistical analyses

SPSS 22 for Mac (SPSS Inc., USA) was used for all analyses, and values are expressed as means ± SEM. Baseline values represent the mean of the first value (*t* = −30 min) and second value (*t* = 0 min) obtained immediately before each hyperinsulinaemic–hypoglycaemic clamp was started. We calculated blood glucose plateaus for all hormones, using mean values of each hormone at clamp time points 30 and 60, 90 and 120, 150 and 180, and 210 and 240 min to obtain four blood glucose plateaus over the entire clamp time of 240 min. We performed ANOVA for key hormones (glucagon, adrenaline and GH) to compare the course of hypoglycaemic counterregulation (‘time’) during sleep and sleep deprivation (‘condition’). We also included *z*-transformed results for key hormones (glucagon, adrenaline and GH) during the lowest blood glucose plateau in a supraordinate ANOVA of *z* scores with the factors hormones, condition and clamp to estimate the general effects of sleep deprivation vs sleep on counterregulation. Subsequently, the results of clamp 3 of the sleep condition were compared with those of the other clamps, i.e. sleep deprivation/clamp 1, sleep deprivation/clamp 3 and sleep/clamp 1 using Helmert contrast tests for orthogonal comparisons to explore first- vs second-level differences (e.g. ‘sleep’ vs ‘sleep deprivation’). A *p* value <0.05 was considered significant.

## Results

### Counterregulatory hormones

Blood glucose concentrations reached the predefined plateaus of 3.75, 3.25, 2.75, and 2.5 mmol/l as intended by the protocol, and were comparable between conditions and clamps (*p*>0.50 for all comparisons, Fig. 1b). Under both conditions, baseline values of glucagon, GH and adrenaline were significantly lower at the start of clamp 3 compared with clamp 1 (*p*<0.026 for the factor ‘clamp’ and respective pairwise comparisons) in the absence of differences between conditions (*p*>0.111 for respective interactions between the factors ‘condition’ and ‘clamp’ and respective pairwise comparisons). The other hormonal baseline values before the respective clamp procedures did not differ between conditions or clamps (*p*>0.219 for all comparisons).

**Fig. 2:**
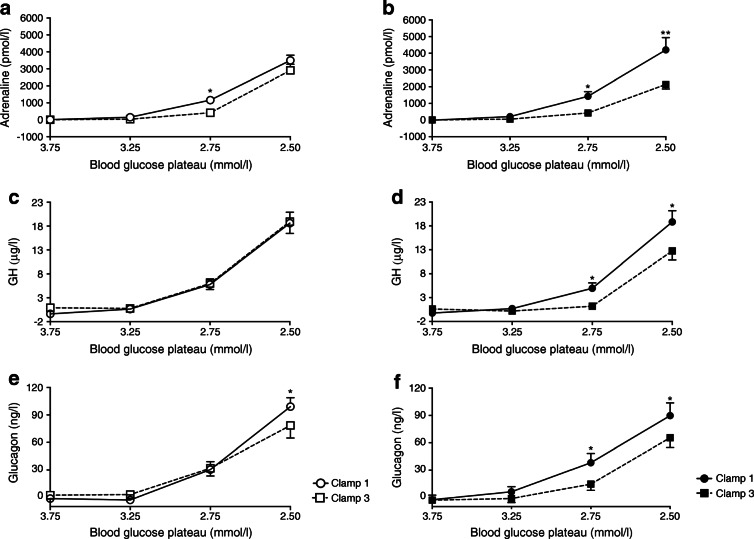
Counterregulatory responses (difference from baseline) for adrenaline (**a**, **b**), GH (**c**, **d**) and glucagon (**e**, **f**) in the sleep deprivation condition (**a**, **c**, **e**) and sleep condition (**b**, **d**, **f**) during the target hypoglycaemic plateaus of 3.75, 3.25, 2.75 and 2.50 mmol/l. Solid lines and open or closed circles represent concentrations during clamp 1; dashed lines and open or closed squares represent concentrations during clamp 3. Asterisks indicate statistically significant differences between clamp 1 and clamp 3: *p≤0.05; **p≤0.01

**Fig. 3:**
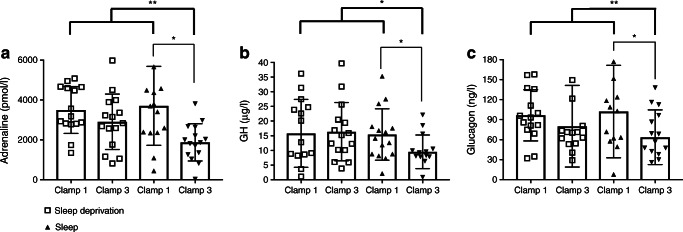
Maximal counterregulatory responses (difference from baseline) for adrenaline (**a**), GH (**b**) and glucagon (**c**) at the target blood glucose plateau of 2.5 mmol/l. Asterisks indicate statistically significant differences: *p≤0.05; **p≤0.01. Bold lines represent the Helmert contrast tests for orthogonal comparisons (clamp 3 of the sleep condition was compared with those of the other clamps, i.e. sleep deprivation/clamp 1, sleep deprivation/clamp 3 and sleep/clamp 1). Faint lines show *t* tests

Sleep deprivation compared with regular sleep dampened the neuroendocrine adaptation to recurrent hypoglycaemia. During hypoglycaemia, the increase in the concentration of adrenaline was attenuated during clamp 3 in the sleep condition, but maintained after sleep deprivation (*F*(1.0;14.0) = 8.142, *p*=0.004, for the interaction between the factors ‘condition’ and ‘time’; Fig. 2a,b). This pattern was also found for GH (*F*(1.0;14.0) = 3.017, *p*=0.089; Fig. 2c,d). While glucagon concentrations appeared less affected (*F*(1.0;14.0) = 2.201, *p*=0.141, Fig. 2e,f), the overall analysis of the key hormones adrenaline, GH and glucagon largely corroborated the attenuating effect of sleep deprivation on adaptation to recurrent hypoglycaemia (*F*(1.0; 14.0) = 4.049, *p*=0.064). Thus, the maximum increases in adrenaline, GH and glucagon were weaker during clamp 3 compared with clamp 1 in the sleep condition (all *p*<0.03, paired *t* tests; Fig. 3a−c) but did not differ between clamps 1 and 3 in the sleep deprivation condition (all *p*>0.22). Adrenaline, GH and glucagon concentrations were lower during clamp 3 of the sleep condition compared with all other clamps (all *p*<0.02, Helmert contrasts; Fig. 3a−c).

**Fig. 4:**
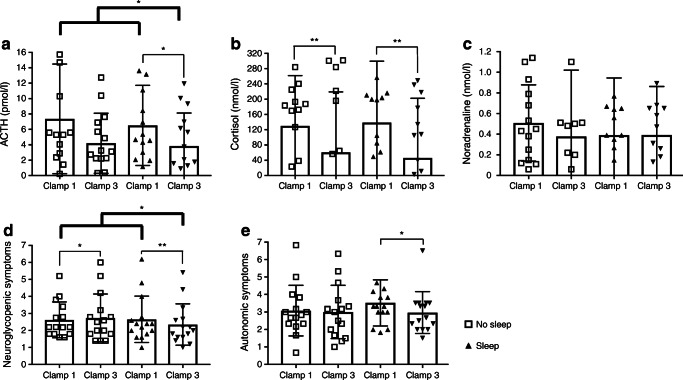
Maximal counterregulatory responses of ACTH (**a**), cortisol (**b**) and noradrenaline (**c**) at the target blood glucose plateau of 2.5 mmol/l. Counterregulatory responses (difference from baseline) in terms of neuroglycopenic (**d**) and autonomic symptoms (**e**) (reported as symptom score, as detailed in the Methods) at the target blood glucose plateau of 2.5 mmol/l. Asterisks indicate statistically significant differences: *p≤0.05; **p≤0.01. Bold lines represent the Helmert contrast tests for orthogonal comparisons (clamp 3 of the sleep condition were compared with those of the other clamps, i.e. sleep deprivation/clamp 1, sleep deprivation/clamp 3 and sleep/clamp 1). Faint lines show *t* tests

The pattern of sleep deprivation effects was less pronounced but still discernible for ACTH concentrations. The decrease in ACTH concentrations during the maximal counterregulatory response between clamp 1 and clamp 3 in the sleep condition was significant (*p*=0.04, paired *t* test; Fig. 4a), but not in the sleep deprivation condition (*p*=0.06, paired *t* test; Fig. 4a). Cortisol concentrations showed a reduction in clamp 3 in both conditions (each *p*<0.005, paired *t* tests; Fig. 4b). There was no decrease in noradrenaline concentrations between clamps 1 and 3 in any condition (*p*>0.46; paired *t* tests; Fig. 4c).

### Neuroglycopenic and autonomic symptoms

The pattern of subjective hypoglycaemic symptoms also reflects a distinct influence of sleep deprivation on the attenuation in response to recurrent hypoglycaemia. After regular sleep, neuroglycopenic symptoms were attenuated during recurrent hypoglycaemia compared with the sleep deprivation condition (*p*<0.05, ANOVA). Neuroglycopenic symptoms were strongly reduced during clamp 3 vs clamp 1 in the sleep condition (*p*=0.005, paired *t* tests; Fig. 4d), but increased after a night of sleep deprivation (*p*=0.014, paired *t* tests; Fig. 4d). Autonomic symptoms also decreased after a night of sleep (*p*=0.019, paired *t* tests; Fig. 4e) but were unchanged after sleep deprivation (*p*=0.859, paired *t* tests; Fig. 4e).

### Sleep variables

Sleep duration and polysomnographic results were in accordance with usual laboratory sleep recordings in healthy young men (Table 1).

**Table 1:** Sleep variables and polysomnographic results under sleep condition

Sleep variables	Duration (min)	Percentage of total sleep time
Total sleep duration	470.6±2.5	100
Wake time after sleep onset	10.2±3.3	2.2 ± 0.7
Sleep stage 1	19.4±4.3	4.1 ± 0.9
Sleep stage 2	260.7±11.0	55.4 ± 2.4
SWS	104.9±10.3	22.3 ± 2.1
REM sleep	73.6±6.2	15.6 ± 1.3
Movement arousal/movement artifacts	1.8±0.4	0.4 ± 0.1

Data are means ± SEM

## Discussion

Sleep is known to support processes of memory formation [7], and, moreover, to contribute to glucose homeostasis [12, 13]. Against this background, we investigated the effect of one night of total sleep deprivation on the adaptation of the counterregulatory response to recurrent hypoglycaemia. In line with our hypothesis, sleep enhanced the adaptation of counterregulatory responses to hypoglycaemia, or, conversely, sleep deprivation prevented the adaptation of key neuroendocrine components of the counterregulatory response and acted to preserve hypoglycaemia awareness in healthy participants. These findings suggest that sleep drives the consolidation of an adaptation in neurometabolic memory, and is a potential target for interventions to improve hypoglycaemia awareness in the clinical context.

Our model of recurrent hypoglycaemia proved to be effective because the psychoneuroendocrine response to low blood glucose levels was distinctly suppressed following two hypoglycaemic episodes in the control condition. In line with previous studies [17], concentrations of glucagon, adrenaline, GH, ACTH and cortisol as well as neuroglycopenic and autonomic symptoms were distinctly lower during clamp 3 than clamp 1, when regular sleep occurred before clamp 3. These changes in the secretory activity of the neuroendocrine stress axis and in subjective awareness reflect an adaptation process of the central nervous system to recurrent substrate deficiency that may recruit brain areas such as the hypothalamus, hippocampus and amygdala that are strongly involved in the control of metabolic processes such as thermogenesis and glucose homeostasis [19] and also in memory processes [7]. During hypoglycaemia, patients with diabetes and hypoglycaemia unawareness show a reduced uptake of [^18^F]-fluorodeoxyglucose to relevant brain regions including hypothalamic nuclei, the left amygdala and the bilateral ventral striatum [20]. Adaptation of hypoglycaemia counterregulation as a process of neurometabolic memory formation may be assumed to recruit a broad network of brain nuclei involved in the regulation of glucose homeostasis and memory consolidation.

In this study, two hypoglycaemic episodes during clamp 1 and clamp 2 were performed to trigger hypoglycaemia unawareness, i.e. the clinically well-known adaptation of the neuroendocrine response to subsequent hypoglycaemia during clamp 3. Our present findings indicate that sleep is a prerequisite for the emergence of robust counterregulatory adaptation to recurrent hypoglycaemia, and are in agreement with convincing evidence for a close interaction of sleep, wakefulness and glucose homeostasis [12, 21, 22]. Sleep not only modulates the response to (experimentally induced) hypoglycaemia during sleep itself [23], but also to a hypoglycaemic episode the following morning. Thus, basal morning concentrations of glucagon were reduced after one night of sleep deprivation compared with regular sleep [14], suggesting that sleep deprivation does not exert an acute stimulatory effect on glucagon secretion as would be expected during an acute stress reaction [24]. Sleep deprivation also enhanced the dynamic counterregulatory responses to hypoglycaemia of glucagon and, to a lesser extent, GH concentrations in our previous study [14], suggesting that direct effects of sleep deprivation on hypoglycaemia counterregulation may add to sleep deprivation-dependent changes in the counterregulatory adaptation. However, such direct effects of sleep deprivation on the counterregulatory response are relatively modest given that sleep deprivation does not substantially attenuate neuroglycopenic and autonomic symptoms during subsequent hypoglycaemia compared to sleep (Fig. 4) [25]. Moreover, and in line with previous studies [14, 26, 27], we did not detect a direct stimulatory effect of sleep deprivation on morning activity of the hypothalamic–pituitary–adrenal axis compared with regular sleep. Accordingly, sleep deprivation probably does not trigger a novel stressor that dishabituates the neuroendocrine adaptation to recurrent hypoglycaemia [28, 29].

Sleep is commonly recognised as an important mediator of memory consolidation [7] in the psychological and immunological domains [30, 31]. Sleep also consolidates acquired memories with adverse and disease-supporting content [32]. However, systematic investigations into the contributions of sleep to neurometabolic memory formation are still scarce. In the psychiatric context, impairing the consolidation of traumatic experiences by sleep deprivation or sleep curtailment might prevent the development of post-traumatic stress disorders [33, 34]. Sleep, and especially deep slow-wave sleep (SWS), has been shown to play a crucial role for neurobehavioural function and sleep-related memory consolidation [35]. The occurrence of nocturnal SWS is associated with metabolic, hormonal and neurophysiological changes that are expected to distinctly alter the activity of hypothalamic regulatory networks involved in glucose homeostasis. SWS is accompanied by decreased overall brain glucose utilisation [36], but selectively increases hippocampal glucose utilisation due to increased neuronal activity [11]. Declarative memory formation during sleep has been shown to depend especially on hippocampal processing and transfer into neocortical neuronal networks during SWS [9]. This consolidation process is supported by and dependent on neuroendocrine mechanisms at the hypothalamic level [37]. Given the overlap between neuroendocrine pathways (particularly with respect to limbic–hippocampal circuitry) that are crucial for sleep-dependent consolidation of declarative memories and those that establish glucose homeostasis, we assume that the regulation of neurometabolic memory formation at least in part depends on hippocampal processing during SWS. Potential differences in underlying mechanisms notwithstanding, it is tempting to speculate that sleep and its role in the consolidation of metabolic memory for events such as hypoglycaemic episodes might also be a target in the treatment of hypoglycaemia unawareness in patients with diabetes.

Our experiments were performed in a small group of healthy men. Although we do not expect gender differences, we cannot rule out the possibility that the observed effects may be more or less pronounced in women. Moreover, the study should be repeated in patients with diabetes. Future studies are also needed to identify the mechanisms underlying the effect of sleep deprivation on the adaptation to recurrent hypoglycaemia and to single out the contribution of direct effects on counterregulatory processes. Hypoglycaemia unawareness is often observed in clinical practice [38], and elderly patients are at a particular risk because subjective awareness of hypoglycaemia decreases with age despite unchanged neuroendocrine counterregulation [39]. Therefore, improving subjective awareness of hypoglycaemia is key for patients with diabetes to break the vicious cycle of undetected severe hypoglycaemic episodes and the resulting adaptation-induced hypoglycaemia unawareness. Our results indicate that sleep deprivation is able to preserve neuroendocrine counterregulation and subjective awareness in response to recurrent hypoglycaemia in an acute setting. They call for investigations into the role of sleep-related processes in chronic hypoglycaemia unawareness that may open new perspectives for prevention of this debilitating complication of diabetes treatment.

## Data Availability

The datasets generated during and/or analysed during the current study are available from the corresponding author on reasonable request.

## Abbreviations

GH: Growth hormone
REM: Rapid eye movement
SWS: Slow-wave sleep
